# Supplementary material for: Inter-Site Cooperativity of Calmodulin N-Terminal Domain and Phosphorylation Synergistically Improve the Affinity and Selectivity for Uranyl
Source: Biomolecules. 2022 Nov 17;12(11):1703. doi: 10.3390/biom12111703 (PMC9687771; doi:10.3390/biom12111703)
Supplement: Supplementary file 1 [file biomolecules-12-01703-s001.zip › biomolecules-2000806-supplementary.pdf]

SUPPORTING INFORMATION FOR THE PAPER

Inter-site cooperativity of Calmodulin N-terminal domain and phosphorylation synergistically improve the affinity and selectivity for uranyl

Maria Rosa Beccia[1,2], Sandrine Sauge-Merle[1], Nicolas Bremond[1], David Lemaire[1], Pierre Henri[3,4], Christine Battesti[1], Philippe Guilbaud[5], Serge Crouzy[6x], Catherine Berthomieu[1] *

Numerical method developed to retrieve the conditional equilibrium constants from fluorescence and calorimetric experiments

The numerical computation performed in this work aims at producing synthetic fluorescence and calorimetric titration curves, with the objective to perform a best fit routine on experimental fluorescence and calorimetric titration curves. The numerical computation is performed as a 3-step process.

First, the concentrations of species in chemical equilibrium is computed, assuming given conditional equilibrium constants, K1 and K2. This is performed by solving the set of coupled nonlinear equations formed by the mass-balance and reaction equilibrium equations, using an iterative Newton–Raphson method, in a manner similar to what is described in Alderighi *et al.* (1999). Some optimisations have been implemented to ensure consistent and fast convergence. For instance, the convergence efficiency of the Newton-Raphson method requires to start the iteration close enough from the solution, to avoid convergence of the algorithm on spurious local minima. To overpass this limitation, we assume continuity of the numerical solution, initialize the resolution for asymptotically low concentration of one of the reactants (protein or metal, depending on the relevant experiment), for which the asymptotic analytical speciation solutions can be derived. That allows to initialise the Newton-Raphson iterative algorithm and reach the solution using a substep procedure that continuously increases the reactant concentration up to the target concentration and solves the speciation recurrently, through a fast convergence, by using a linear interpolation of the numerical solutions obtained from previous iterations. This first step of our numerical computation has been extensively validated by confrontation to both analytical solutions for known chemical systems and the HySS program.

Second, the synthetic fluorescence (respectively ITC) signal is computed from the free uranyl concentration [M] obtained from the result of the previous speciation computation. This synthetic fluorescence (respectively ITC) curve is a function of both given conditional equilibrium constants, K_1 and K_2, and the assumed amplitude of the binding isotherm $\Delta\phi$ (respectively of the molar enthalpies associated to the binding of the metal ion).

Third, a minimization of the distance between the experimental and synthetic fluorescence (respectively ITC) curves is performed using either a multidimensional gradient descent method, or a full scan of possible parameter. The best fit enables to identify the conditional equilibrium constants, K_1 and K_2, together with by-products such as the binding isotherm $\Delta\phi$ or/and the molar enthalpies associated to the binding of the metal ion.

A specificity of this numerical method, compared to other available in the literature, is that the fluorescence and calorimetry fitting routines are directly embedded into the speciation routine.

Reference

Alderighi L, Gans P, Ienco A, Peters D, Sabatini A, Vacca A, Hyperquad simulation and speciation (HySS): a utility program for the investigation of equilibria involving soluble and partially soluble species. *Coordination Chemistry Review* 1999, 184:311-318.

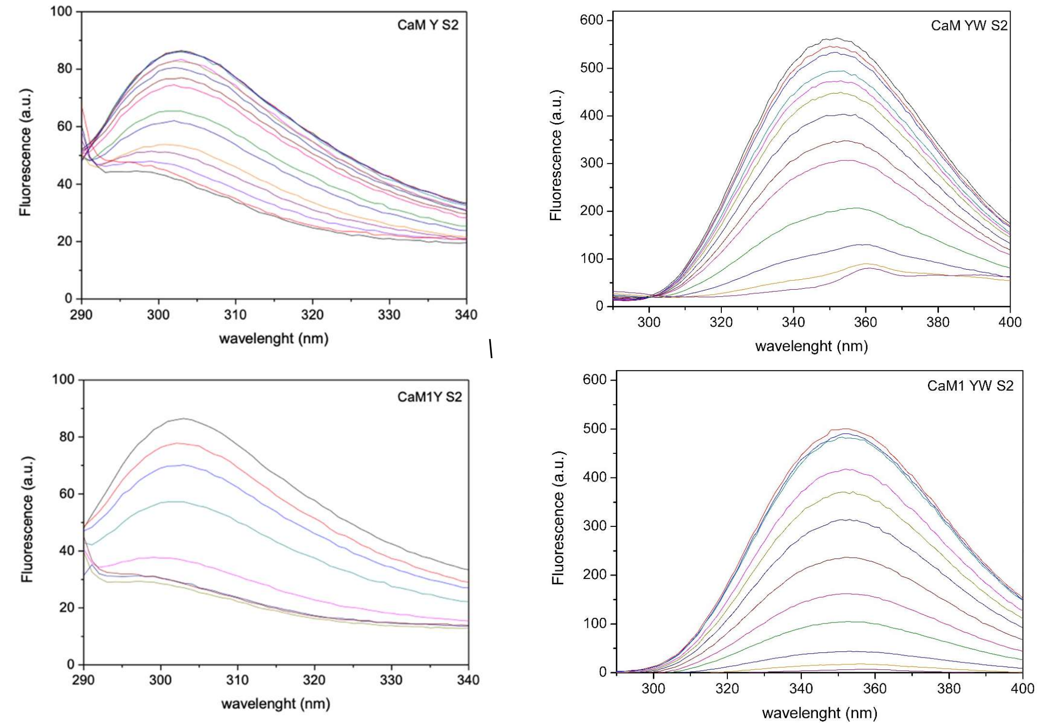

Figure S1. *Fluorescence spectral changes induced by UO_2^{2+} binding to the one-site peptides CaM Y II, CaM1 Y II, CaM YW II and CaM1 YW II. Spectra were recorded during spectrofluorimetric titrations and corrected for the dilution at each point of the titration. pH = 6, T = 298 K.*

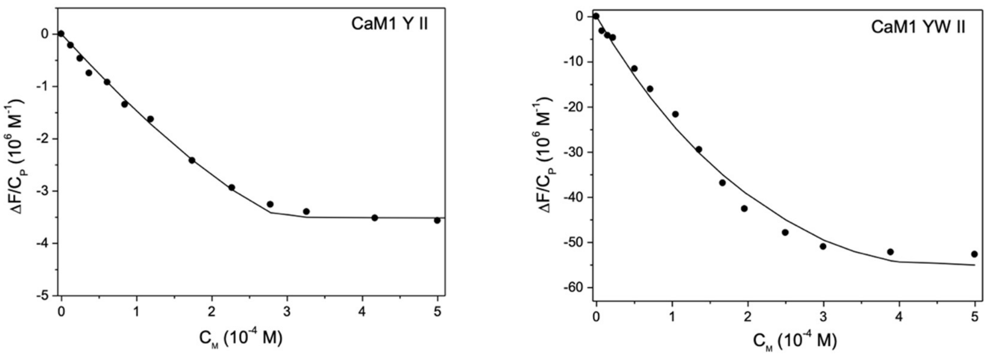

Figure S2. *Fluorescence binding isotherms for the interaction of UO_2^{2+} ion with the one-site peptides CaM1 Y II (at λ = 302 nm) and CaM1 YW II (at λ = 350 nm); initial $C_P = 1.8.10^{-5}$ M, initial $C_I = 3.10^{-4}$ M for CaM1 Y II, initial $C_P = 1.10^{-5}$ M, initial $C_I = 5.10^{-4}$ M for CaM1 Y II. pH = 6, T = 298 K. Experimental data (dots) and adjustment (black solid line), according to equation (5).*

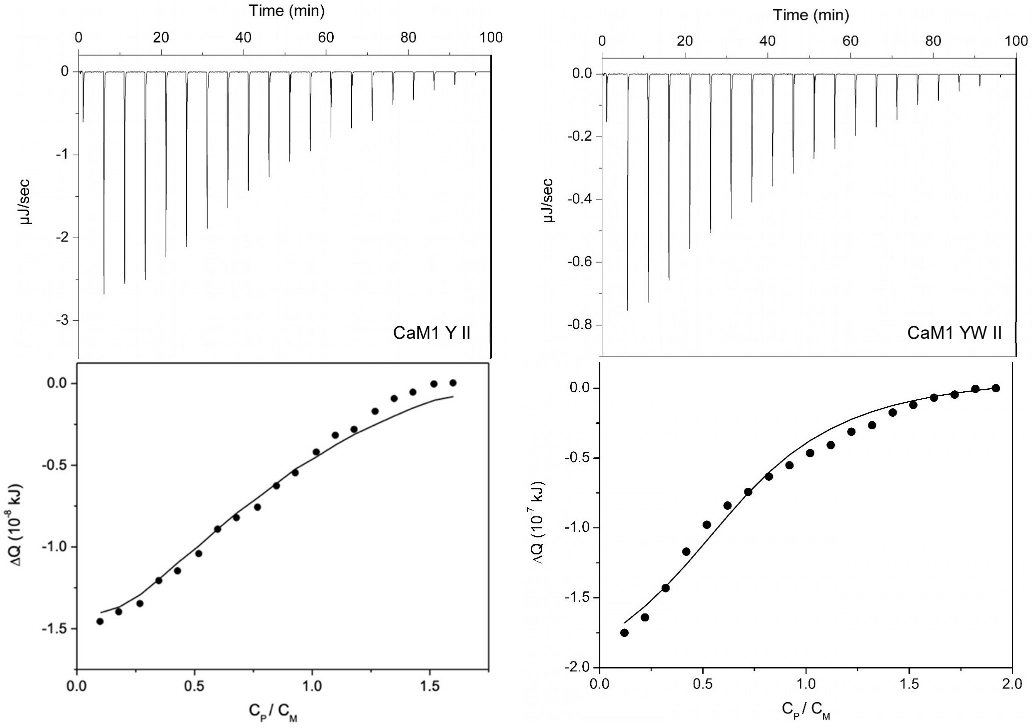

Figure S3. *ITC raw data and binding isotherm for UO_2^{2+}ion interaction with the one-site peptides CaM1 Y II and CaM1 YW II; syringe: $C_P = 2.5.10^{-3}$ M, cell: $C_M = 3.10^{-4}$ M and $C_I = 6.10^{-4}$ M for CaM1 Y II, $C_I = 2.10^{-3}$ M for CaM1 YW II. pH = 6, T = 298 K. Experimental data (dots) and adjustment (black solid line), according to equation (11).*

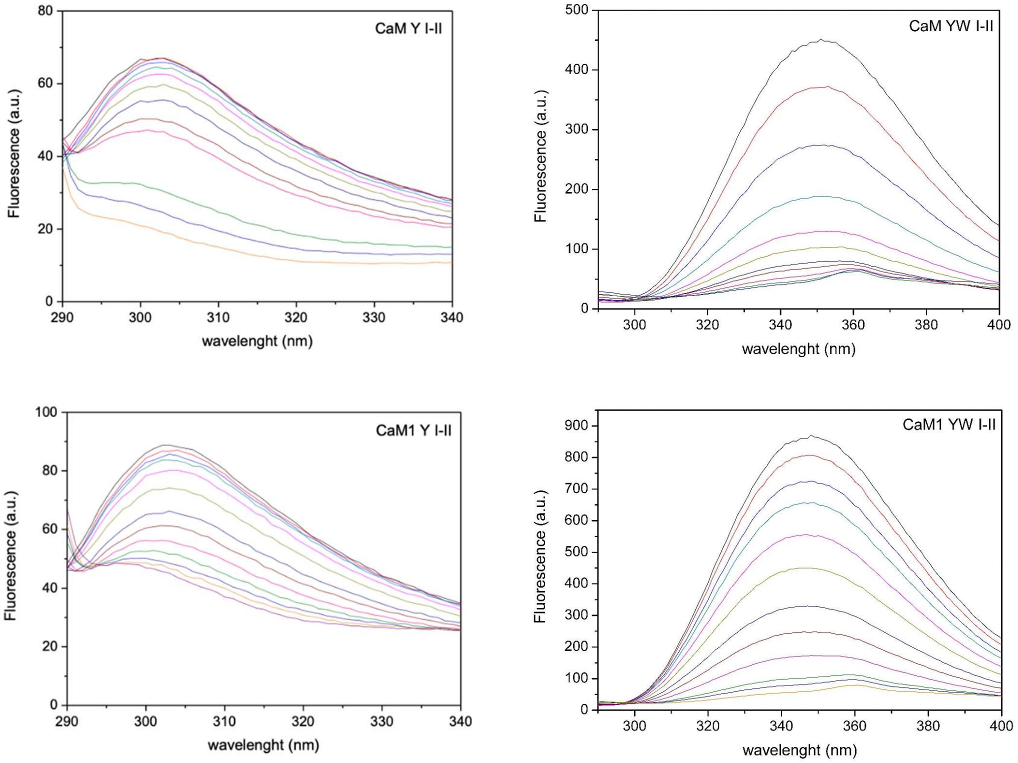

Figure S4. *Fluorescence spectral changes induced by UO_2^{2+} binding to the non-phosphorylated two-site peptides CaM Y I-II, CaM1 Y I-II, CaM YW I-II and CaM1 YW I-II. Spectra were recorded during spectrofluorimetric titrations and corrected for the dilution at each point of the titration. pH = 6, T = 298 K.*

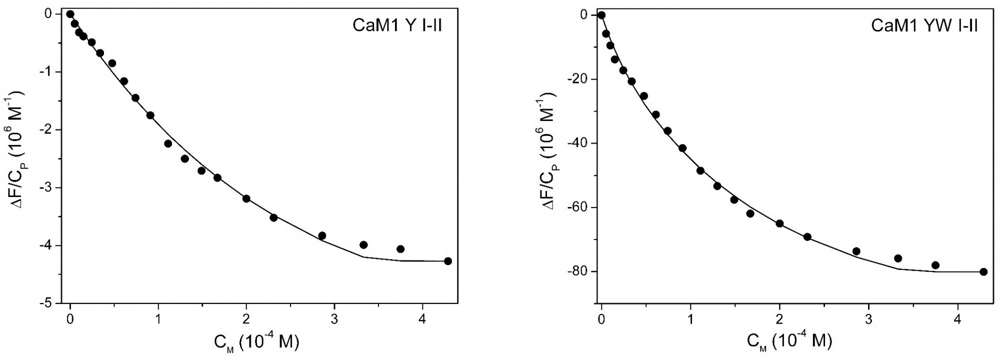

Figure S5. *Fluorescence binding isotherms for the interaction of UO_2^{2+} ion with the non-phosphorylated two-sites peptides CaM1 Y I-II (at λ = 302 nm) and CaM1 YW I-II (at λ = 350 nm); initial $C_P = 1.10^{-5}$ M, initial $C_I = 5.10^{-4}$ M, pH = 6, T = 298 K. Experimental data (dots) and adjustment (black solid line), according to equation (7).*

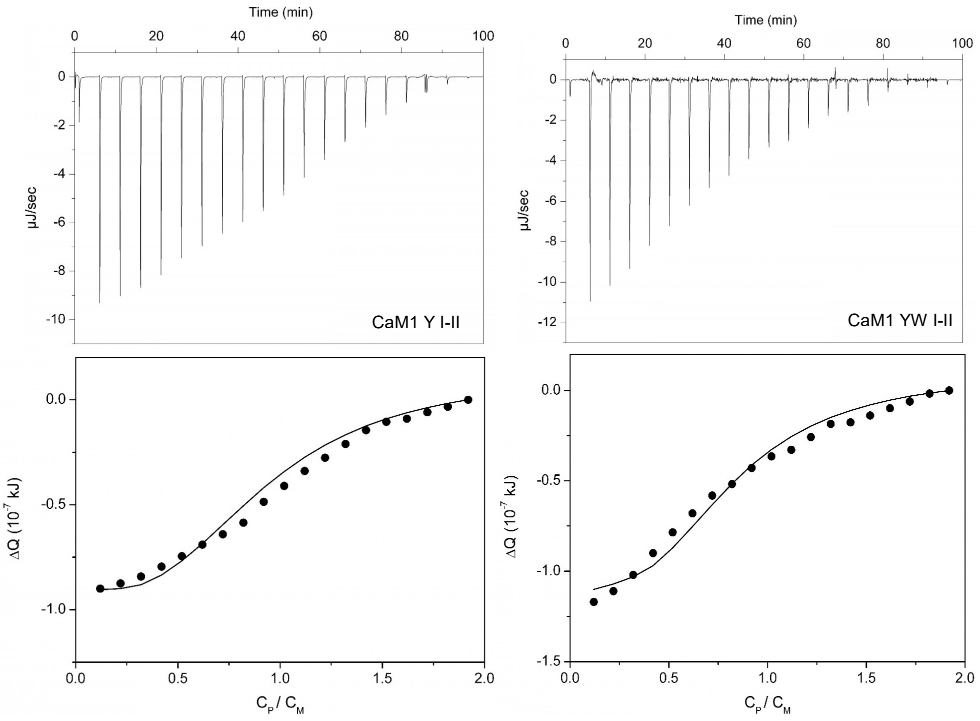

Figure S6. *ITC raw data and binding isotherm for UO_2^{2+} interaction the non-phosphorylated two-sites peptides CaM1 Y I-II and CaM1 YW I-II; $C_P = 2.10^{-3}$ M (syringe), $C_I = 4.10^{-4}$ M and $C_M = 2.10^{-4}$ M (cell) pH = 6, T = 298 K. Experimental data (dots) and adjustment (black solid line), according to equation (16).*

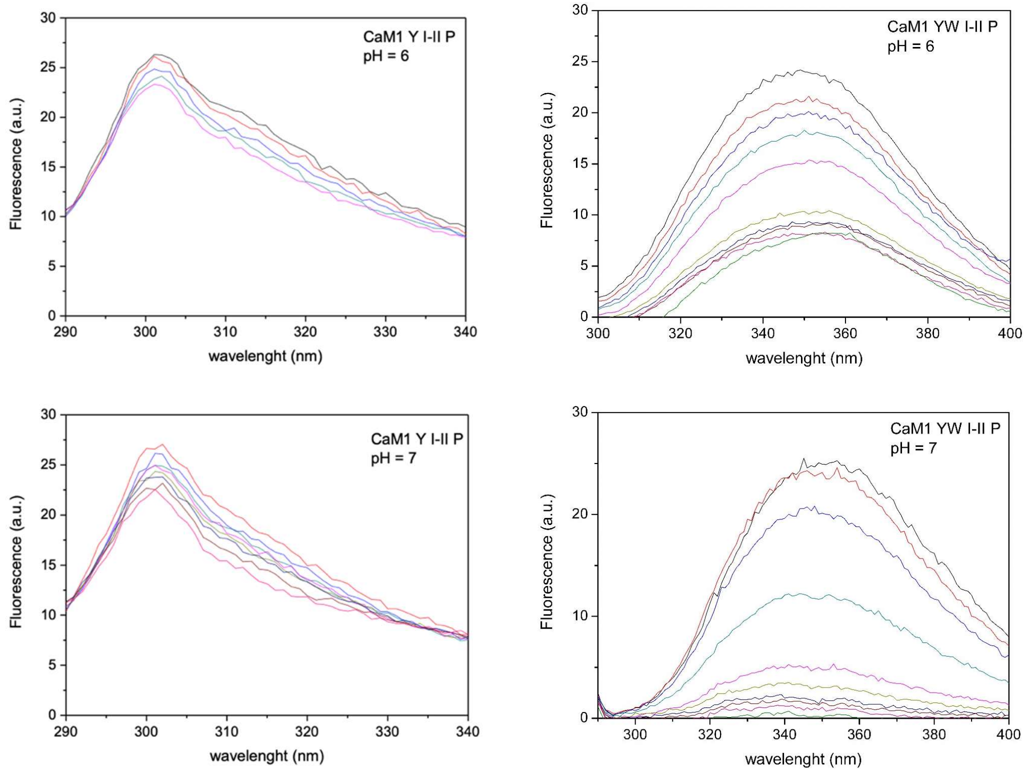

Figure S7. *Fluorescence spectral changes induced by UO_2^{2+} binding to the phosphorylated two-site peptides CaM1 Y I-II P and CaM1 YW I-II P at pH 6 and pH 7. Spectra were recorded during spectrofluorimetric titrations and corrected for the dilution at each point of the titration. T = 298 K.*

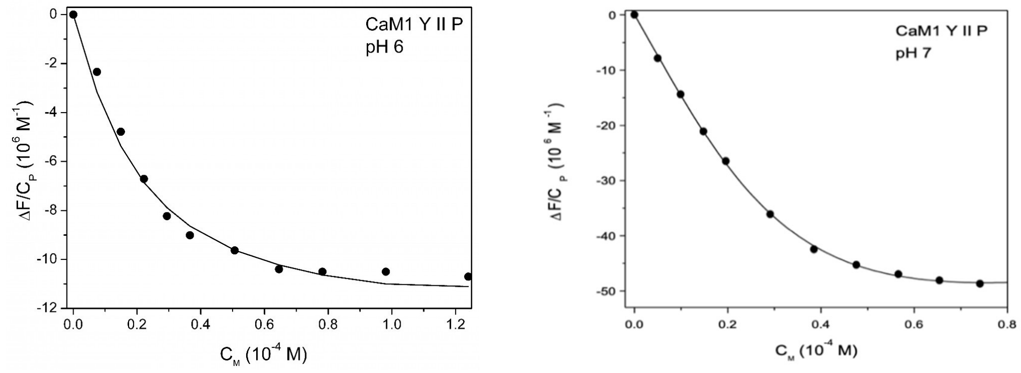

Figure S8. *Binding isotherms for the interaction of UO_2^{2+} with the phosphorylated one-site peptide CaM1 Y II P at pH 6 and pH 7 (at λ = 302 nm); initial $C_P = 1.10^{-5}$ M, initial $C_I = 1.10^{-4}$ M, T = 298 K. Experimental data (dots) and adjustment (black solid line), according to equation (5).*

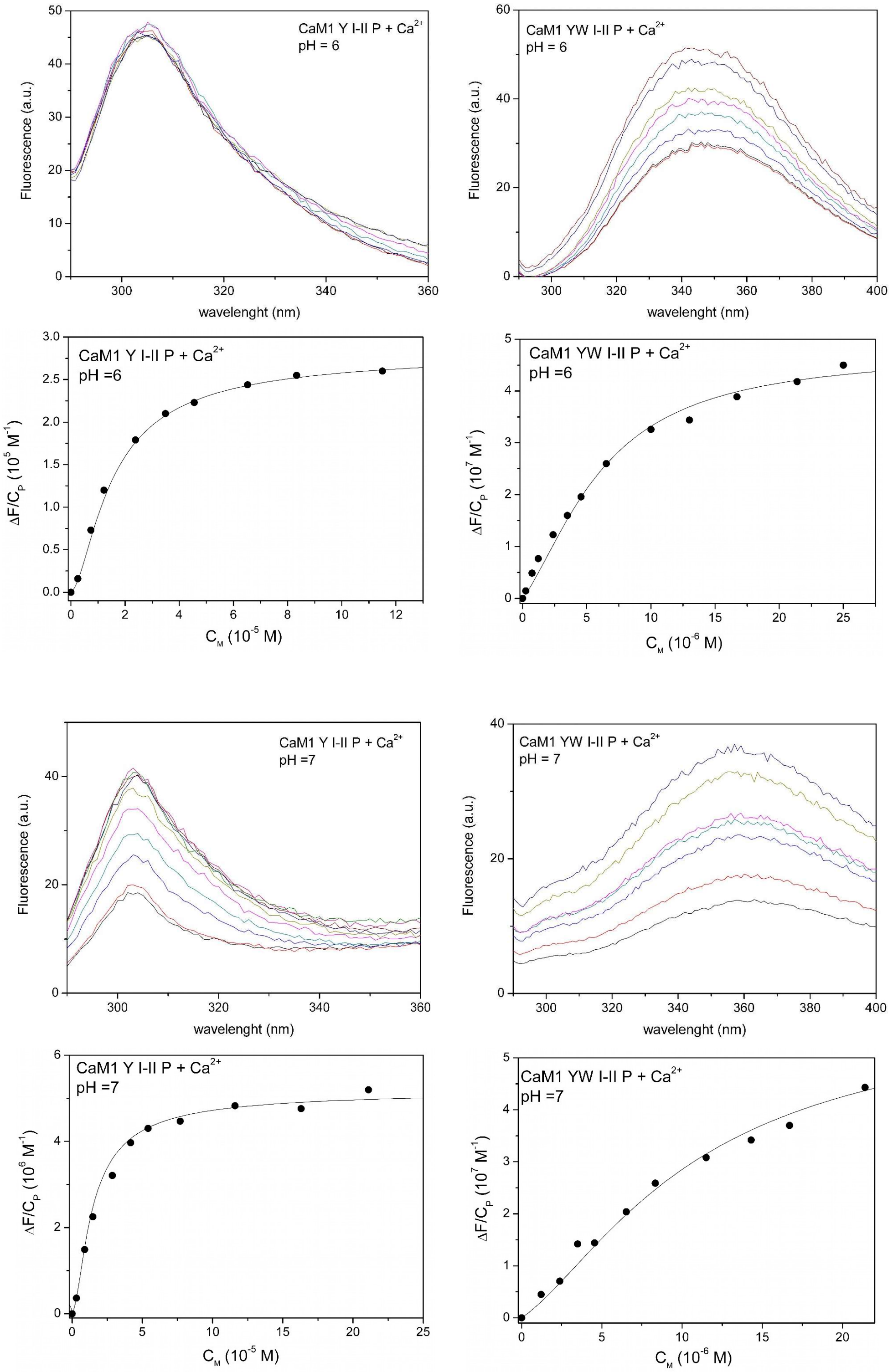

Figure S9. *Fluorescence spectral changes and corresponding binding isotherm for the interaction of Ca^{2+} ion with the phosphorylated two-sites peptide CaM1 Y II P (at λ = 302 nm) and CaM1 YW II P (at λ = 302 nm); experiments were performed without IDA. pH = 6 for top graphs and pH = 7 for lower graphs, T = 298 K.. Experimental data (dots) and adjustment (black solid line), according to equation (7).*

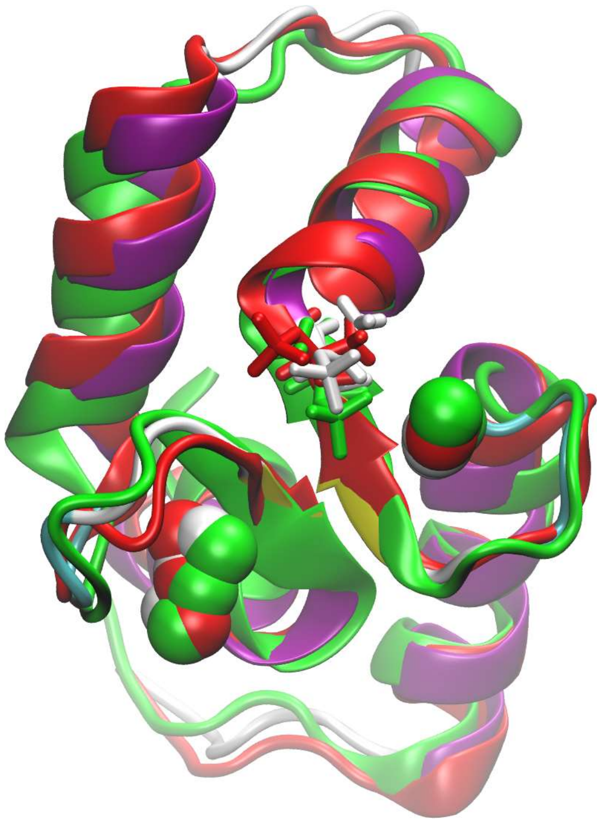

Figure S10. *Superimposition of initial structure of CaM1 Y I-II P (white, yellow magenta) with the structures obtained after 12 ns NPT MD using AMBER, with (green) or without (red) introduction of distance restraints during 200 ps prior to the simulation.*

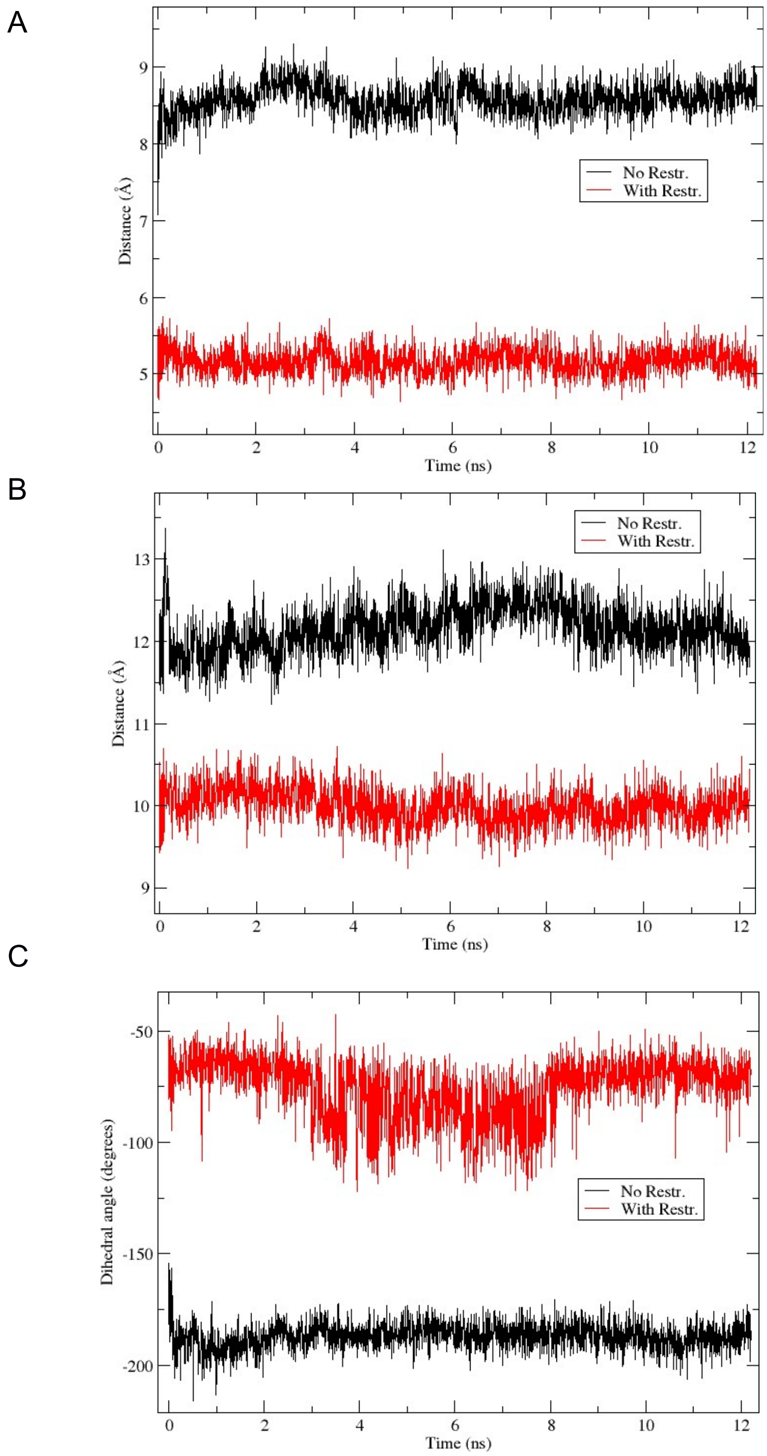

Figure S11. *Evolution of A) the P-U1 distance, B) the P-U2 distance, and C) the N-Cα-Cβ-Oγ dihedral angle of Thr9 of site I with time for the 12 ns NPT MD simulation performed with Amber using the ff14SB force field, with (*"With Restr."*, red lines) or without (*"No Rest.*", black lines) introduction of distance restraints during 200 ps MD prior to the simulation.*

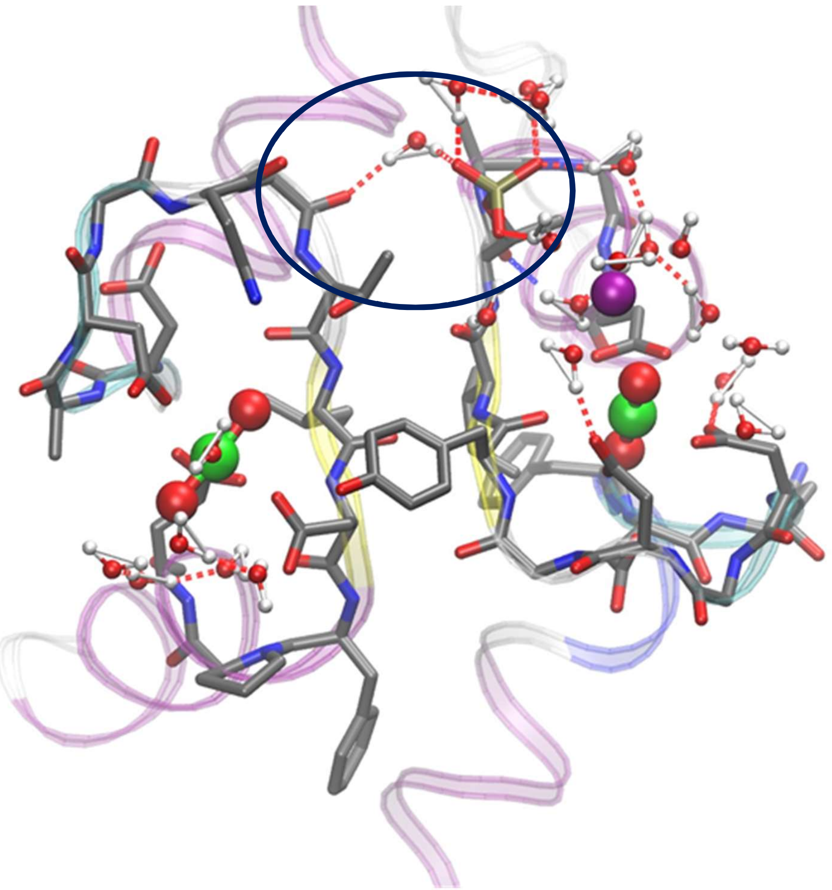

Figure S12 : *Structural model of the CaM1 Y I-II P – (UO_2^{2+})$_2$ complex obtained after 12 ns NPT MD using a polarized force field, with (green) or without (red) introduction of distance restraints during 200 ps prior to the simulation. The hydrogen-bonding network involving a water molecule between one phosphoryl oxygen of site I Thr9$_P$ and the peptide carbonyl oxygen of site II Gly6 is highlighted in the circled part of the figure.*

Table S1. *Primers used to generate the peptide variants. The primers were synthesized according to E. coli codon usage.*

Goal	Sequences (5' → 3')
[1]Mutations Asp1Ala and Asp3Ala-S	TTTCAGCTTATTCGCCAAGGCTGGTGATGGTTAC
[1]Mutations Asp1Ala and Asp3Ala-AS	GTAACCATCACCAGCCTTGGCGAATAAGCTGAA A
[2]Mutations Thr10Ala and Lys11Ala-S	GGTGATGGTTACATTACCGCCGCGGAGCTGGGTACTGTGATGCG
[2]Mutations Thr10Ala and Lys11Ala-AS	CGCATCACAGTACCCAGCTCCGCGGCGGTAATGtAACCATCACC
[3]Mutation Thr7Trp-S	GATGCTGATGGTAACGGTTGGATTGATTTCCCAGAGTTC
[3]Mutation Thr7Trp-AS	GAACTCTGGGAAATCATTCCAACCGTTACCATCAGCATC
D1-CaM-TEV-S	GAGAGGATCCGAGAACCTGTACTTCCAGTCCATGGCGGATCAGCTCACCGACGAT
D1-CaM-STOP-AS	GAGAGAGAGAGAGACTGCAGTCACTTACGAGCCATAAGGTTCAAGAAC

[1]Mutations impair site 1 and prevent metal binding; [2]Mutations generate the TEV protease recognition site in site I; [3]The mutation allows the introduction of a Trp residue at position 7 in site II to control the fluorescence intensity.

Table S2*. Geometric parameters obtained for the distances between site I Thr9$_P$ P and the uranium atom of UO_2^{2+} present at site I (U1) and at site II (U2) and for the dihedral angle of Thr9$_P$ side-chain in the structural model resulting from the 15 ns MD simulation without restraints (No-Restr.) or starting with a structure with distance restraints (With Restr.)*

Geometric Parameters.	"No-Restr."		"With-Restr".	
P-U1 (Å)	8.58	± 0.18	5.17	± 0.16
P-U2 (Å)	12.25	± 0.27	9.94	± 0.20
N-Cα-Cβ-Oγ (°)	-187.0	± 5.5	-76.0	± 13.6
